# Effects of urolithin A on osteoclast differentiation induced by receptor activator of nuclear factor-κB ligand via bone morphogenic protein 2

**DOI:** 10.1080/21655979.2022.2036893

**Published:** 2022-02-14

**Authors:** Zhe Wang, Guobin Qi, Zhuokai Li, Xu Cui, Shengyang Guo, Yueqi Zhang, Pan Cai, Xiuhui Wang

**Affiliations:** a Department of Orthopedics, Zhongshan Hospital, Fudan University, Shanghai, China; b Department of Orthopedic Surgery, School of Medicine, Johns Hopkins University, Baltimore, MD, USA; c Department of Orthopedics, Shanghai University of Medicine & Health Sciences Affiliated Zhoupu Hospital, Shanghai, China

**Keywords:** RAW264.7 cells, osteoclast, urolithin A, RANKL, BMP2, transcriptome sequencing

## Abstract

Urolithin A (UA) is an intestinal microbial metabolite derived from ellagitannins and a promising agent for treating osteoarthritis. However, its effects on osteoporosis are unclear. This study explored the effects of urolithin A (UA) on receptor activator of nuclear factor-κB ligand (RANKL)-induced osteoclasts and its underlying molecular mechanisms. RANKL treatment significantly increased tartrate-resistant acid phosphatase (TRACP) or osteoclast marker levels (*P* < 0.05), while adding UA decreased the RANKL-induced levels (*P* < 0.05) in RAW264.7 cells. Total RNA isolated from RANKL- or RANKL + UA-treated cells was sequenced, and the obtained transcriptome dataset revealed 2,399 differentially expressed genes. They were enriched in multiple pathways involved in rheumatoid arthritis, ERK1 and ERK2 cascade, regulation of inflammatory response, ECM-receptor interactions, and TNF signaling. Scanning electron microscopy showed that RANKL promoted bone resorption pits in bone biopsy specimens, whereas UA inhibited their formation. When bone morphogenic protein 2 (BMP2) was *shRNA*-silenced, the bone resorption pits were restored. Moreover, while RANKL significantly enhanced the levels of p-ERK2/ERK2, p-p38/p38, p-Akt1/Akt1, p-ERK1/ERK1, and osteoclast-related proteins (*P* < 0.05), UA reduced them. BMP2 silencing also reversed the UA inhibitory effect. Thus, UA represses the RANKL-induced osteoclast differentiation of RAW264.7 cells by regulating Akt1, p38, and ERK1/2 signaling, and BMP2 likely reverses the UA inhibitory effect via these pathways. We propose BMP2 as a potential drug target for treating bone metabolic diseases, such as osteoporosis.

## GRAPHICAL ABSTRACT

**Figure:**
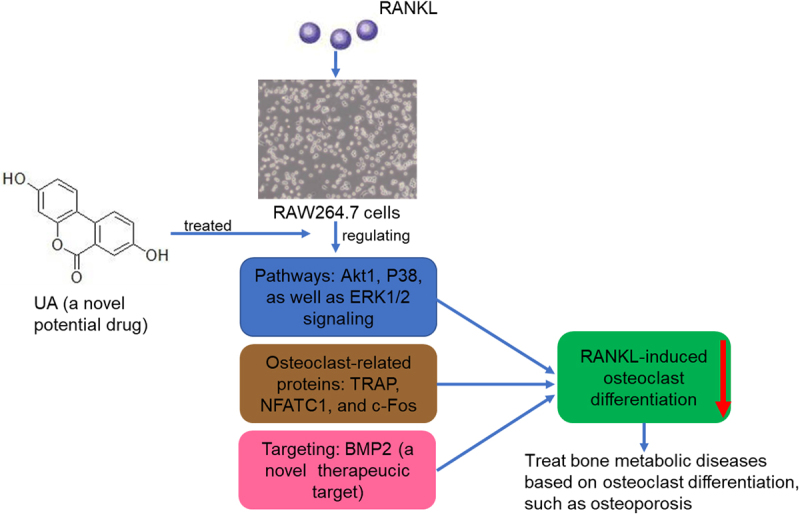

## Background

Bone remodeling involves the dynamic coupling of osteoblast bone formation and osteoclast bone resorption[1]. Under normal conditions, osteoblasts and osteoclasts coordinate to establish homeostasis between the two processes, maintaining normal bone function[2]. Under disease conditions, such as rheumatoid arthritis, osteoporosis, and lytic bone metastases, excessive bone resorption causes imbalance leading to bone loss[3]. Osteoporosis is a life-threatening medical problem that increases with prolonged human life expectancy [4,5]. It often leads to fractures and other complications, such as prolapse pneumonia, deep vein thrombosis, and bedsores, which can be fatal[6]. Currently, the main agents used to treat osteoporosis are antiresorptive (e.g., bisphosphonates, denosumab, and calcitonin) and anabolic (strontium ranelate)[7]. However, their efficacy is unsatisfactory and long-term use can lead to numerous adverse effects. Therefore, discovering new therapeutic strategies to manage osteoporosis is critical.

Urolithin A (UA) is an intestinal microbial metabolite of ellagic acid and ellagitannins. It has high bioavailability and multiple health benefits, including anti-inflammatory and anti-proliferative effects against cancer, antioxidant activity, and improved lipid metabolism [8,9]. A first-in-human clinical trial demonstrated that UA is safe and can induce molecular signatures of improved mitochondrial and cellular health following regular oral consumption[10]. Additionally, UA attenuates the inflammatory response and cartilage degradation induced by IL-1β by inhibiting mitogen-activated protein kinase (MAPK)/ nuclear factor kappa B subunit 1NF-κB signaling pathways. Thus, UA is a promising agent for treating osteoarthritis (OA)[11]. Lin et al.[12] indicated that UA promotes mitophagy by protein kinase AMP-activated catalytic subunit alpha 1 (AMPK) signaling activation in tert-butyl hydroperoxide-treated nucleus pulposus cells, inhibiting the treatment-induced mitochondrial dysfunction and apoptosis. It also alleviates the progression of puncture-induced intervertebral disc degeneration in vivo. However, UA effects on osteoporosis and its underlying molecular mechanisms remain unclear.

Osteoclasts are key participants in bone resorption and unique multinuclear cells originating from the mononuclear/macrophage lineage of the bone marrow[13]. Therefore, they may be the targets for osteoporosis treatment[14]. Their differentiation and function are regulated by various systemic hormones, cytokines, and the central osteoclastogenic molecule called receptor activator of nuclear factor-κB ligand (RANKL)[13]. The interaction between RANKL and its receptor (RANK) activates several transcription factors and signaling pathways[15], stimulating the expression of genes that control osteoclast differentiation and functions and promoting osteoclast formation and maturation[16]. Excessive osteoclast activity in osteoporosis causes an imbalance in bone remodeling; therefore, inhibiting it is essential in preventing and treating osteoporosis.

RAW264.7 is a mature pre-osteoclast cell line that can differentiate into osteoclasts after induction by specific growth factors, such as RANKL[17]. RANKL, expressed as a membrane-associated cytokine, has been used to induce osteoclastic differentiation in the RAW264.7 cells[1], as well as RANKL-treated RAW264.7 cells are often used to study osteoclast genesis in vitro. Therefore, in our study, RANKL was used to induce osteoclast differentiation of RAW264.7 cells and UA to treat the differentiated cells. Effects of UA on osteoclast activity were evaluated, and its mechanisms were determined by transcriptome sequencing. The transcriptome analysis showed that bone morphogenic protein 2 (BMP2) mRNA levels were significantly affected by the UA treatment of the differentiated cells. To our knowledge, the members of the BMP family are key mediators of morphogenesis and differentiation in multiple tissues and play important roles in skeletal development[16]. Moreover, BMP2 regulates osteoclasts and osteoblasts and directly promotes RANKL-induced osteoclast differentiation [17,18]. Therefore, BMP2 was further investigated to determine its role in the UA-promoted inhibition of osteoclast formation. Our work suggests a novel drug target for the treatment of osteoporosis.

## Materials and methods

### Cell culture

RAW264.7 cells, a kind of the mouse macrophage cell line, were purchased from the Cell Bank of Type Culture Collection of Chinese Academy of Sciences (Shanghai, China). The cells were cultured in high-glucose Dulbecco’s Modified Eagle Medium (Thermo Fisher Scientific, Waltham, MA, USA) supplemented with 10% fetal bovine serum (Thermo Fisher Scientific) and 100 U/mL penicillin–100 μg/mL streptomycin (Thermo Fisher Scientific). The cells were maintained in a humidified incubator containing 5% CO_2_ at 37°C.

### Determination of optimal UA concentration

A Cell Counting Kit-8 (CCK-8; Beyotime Biotechnology, Beijing, China) was used to assess the optimal concentration of UA for treating RAW264.7 cells before subjecting them to RANKL-stimulated differentiation. The cells were seeded into 96-well plates at a density of 1 × 10^4^ cells/well and cultured. The adhered cells were treated with 0, 0.5, 5, 25, 50, or 100 μM UA (Selleck Chemicals, Houston, TX, USA) and cultured at 37°C for 12, 24, 48, 72, and 96 h. Upon culturing, 10 μL of the CCK-8 reagent was added to each well, and the cells were incubated for another 2 h. The optical density (OD) was measured at 450 nm using a microplate reader (Thermo Fisher Scientific).

### Quantification of tartrate-resistant acid phosphatase (TRACP)

RAW264.7 cells were divided into three treatment groups: control, RANKL, and RANKL + UA groups. Cells in the RANKL and RANKL + UA groups were treated with 100 ng/mL RANKL[18] and 100 ng/mL RANKL + 25 μM UA, respectively. Following 48 h of culturing, cells were lysed using cell lysis buffer (Beyotime Biotechnology), and the lysate was used for TRACP detection using a TRACP content detection kit (Beyotime Biotechnology), according to the manufacturer’s protocol. Briefly, 40 μL of the cell lysate was incubated at 37°C for 10 min, and 160 μL of stop solution was added. A microplate reader was used to measure the OD at 405 nm.

### Transcriptome sequencing and analysis

The RNA samples isolated from the RANKL- and RANKL + UA-treated cells were sequenced at Yanzai Biotechnology Co, Ltd (Shanghai, China) on an Illumina (San Diego, CA, USA) sequencing platform. Clean data were obtained by removing low-quality sequences and adapter contamination. The HISAT2 software (https://ccb.jhu.edu/software/hisat2/index.shtml) was used for mapping, and gene expression levels were determined using the featureCounts software. The DESeq2 algorithm was used to identify differentially expressed genes (DEGs) based on the fold change (FC) cutoff of > 1.5 and false discovery rate (FDR) < 0.05. The DEGs were subjected to gene ontology (GO) and Kyoto Encyclopedia of Genes and Genomes (KEGG) functional analyses. The GO terms and KEGG pathways were considered significantly enriched if *P* < 0.05.

### Acquisition of lentivirus packaging

Using the sequence reported by Fujimoto et al.[19] (GATCCGGGACACCAGGTTAGTGAATTTCAAGAGAATTCACTAACCTGGTGTCCCTTTTTTCTCGAGG), a *BMP2* short hairpin RNA (shRNA) was synthesized by Yanzai Biotechnology Co, Ltd. The pGreenPuro vector was used to construct the *BMP2* shRNA vector, as previously described[20]. RAW264.7 cells were infected with 50 μL of the packaged *BMP2* shRNA vector suspension and cultured for 48 h. After 48 h, the complete medium containing 2 μg/mL puromycin was added to select for stable, transfected sh*BMP2*-RAW264.7 cells[21]. The stably transfected cells were expanded in culture for subsequent experiments. The *BMP2* primer sequences are shown in Table 1. Reverse transcription-quantitative PCR (RT-qPCR) evaluated the transfection efficiency by determining the *BMP2* expression.

**Table 1.** The sequences of all primers

Gene	Sequence (5’-3’)
TRAP	F: CACTCCCACCCTGAGATTTGT
	R: CCCCAGAGACATGATGAAGTCA
NFATC1	F: GGAGAGTCCGAGAATCGAGAT
	R: TTGCAGCTAGGAAGTACGTCT
c-Fos	F: CGGGTTTCAACGCCGACTA
	R: TGGCACTAGAGACGGACAGAT
BMP2	F: GGGACCCGCTGTCTTCTAGT
	R: TCAACTCAAATTCGCTGAGGAC
Cebpa	F: CAAGAACAGCAACGAGTACCG
	R: GTCACTGGTCAACTCCAGCAC
Clec4a3	F: CTTCACTTCAACTGACTTGGTGG
	R: TCACTGCTAGGCTCACCTTTG
Mapk1ip1	F: CCCTCCAGCTAGACTACTACCA
	R: TGGAAAGGGCACATTTGTTGAAA
MMP3	F: ACATGGAGACTTTGTCCCTTTTG
	R: TTGGCTGAGTGGTAGAGTCCC
MMP9	F: GCAGAGGCATACTTGTACCG
	R: TGATGTTATGATGGTCCCACTTG
RUNX2	F: ATGCTTCATTCGCCTCACAAA
	R: GCACTCACTGACTCGGTTGG
RUNX3	F: CAGGTTCAACGACCTTCGATT
	R: GTGGTAGGTAGCCACTTGGG
GAPDH	F: GGTGAAGGTCGGTGTGAACG
	R: CTCGCTCCTGGAAGATGGTG

### RT-qPCR

Total RNA was extracted from RAW264.7 or sh*BMP2*-RAW264.7 cells after the different chemical treatments using RNAiso Plus (Takara). The RNA quality and concentration were assessed using the 260/280 OD ratio. The isolated RNA with a high 260/280 ratio was reverse-transcribed into cDNA using PrimeScript RT Master Mix (Takara) and used as the template for RT-qPCR. The PCR assays were performed using SYBR Premix Ex Taq II (Tli RNaseH Plus, Takara) in an ABI 7500 detection system (Applied Biosystems, Foster City, CA, USA). The qPCR cycling conditions were as follows: 50°C, 2 min; 95°C, 2 min; 40 cycles at 95°C, 15s; 40 cycles at 60°C, 60s. All primer sequences are listed in Table 1. Glyceraldehyde-3-phosphate dehydrogenase (*GAPDH*) was used as a housekeeping gene, and the relative mRNA expression levels were calculated using the 2^−ΔΔCt^ method[22].

### Scanning electron microscopy

Bone biopsies (IDS Imaging Development Systems Inc, Obersulm, Germany) were washed with medium and placed in 96-well plates. shNC-RAW264.7 cells (negative control) or sh*BMP2*-RAW264.7 cells were seeded into the wells containing the biopsies. The biopsies with shNC-RAW264.7 or sh*BMP2*-RAW264.7 cells were treated with 100 ng/mL RANKL or 100 ng/mL RANKL and 25 μM UA. Subsequently, the treated biopsy specimens were harvested and fixed with 2.5% glutaraldehyde. The images of the bone biopsies were obtained using a scanning electron microscope (Hitachi, Chiyoda City, Japan) and quantified using the NIH ImageJ software (Bethesda, MD, USA).

### Western blotting

Total protein was isolated from RAW264.7 or sh*BMP2*-RAW264.7 cells subjected to different treatments using radioimmunoprecipitation assay lysis buffer (Beyotime Biotechnology). Protein concentrations were determined using a bicinchoninic acid assay kit (Beyotime Biotechnology). The protein samples (20 μg) were separated on a 10% sodium dodecyl sulfate-polyacrylamide gel, transferred to polyvinylidene fluoride membranes, and blocked with 5% skim milk at 37°C for 2 h. After washing with PBST (1 mL of Tween-20 in 1,000 mL of PBS) three times, the membranes were incubated with anti-Akt1 (1:1,000; Cell Signaling Technology, Danvers, MA, USA), anti-phosphorylated (p)-Akt1 (1:1,000; Cell Signaling Technology), anti-P38 (1:1,000; Cell Signaling Technology), anti-p-P38 (1:1,000; Cell Signaling Technology), anti-ERK (1:1000; Cell Signaling Technology), anti-phospho-ERK (1:1,000; Cell Signaling Technology), anti-RANK (1:1,000; Abcam, Cambridge, UK), anti-cathepsin K (1:1,000; Proteintech, Rosemont, IL, USA), anti-integrin β3 (1:1,000; Abcam), or anti-GAPDH (1:1,000, Proteintech) antibodies at 4°C overnight. After washing with PBST three times, the membranes were incubated with the secondary antibody (1:5,000; Jackson ImmunoResearch Laboratories Inc, West Grove, PA, USA) at 37°C for 2 h. After washing the membranes with PBST five times, they were subject to an enhanced chemiluminescence assay (Beyotime Biotechnology). The protein bands were detected in a chemiluminescence imaging apparatus (Shanghai Tanon Science & Technology Co Ltd, Shanghai, China).

### Statistical analysis

Data are reported as the mean ± standard deviation. GraphPad Prism 5 (GraphPad Prism Software, San Diego, CA, USA) was used for statistical analyses. A Student’s *t*-test was used to compare two groups, and a one-way analysis of variance to compare more than two groups. *P* < 0.05 was considered statistically significant.

## Results

### Determination of optimal UA concentration

**Figure 1.**
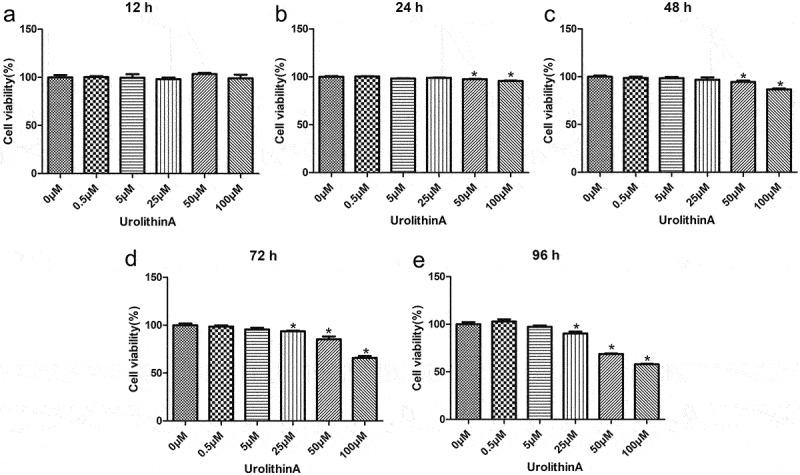
Determination of optimal urolithin A concentration for cell treatments. The viability of RAW264.7 cells treated with different concentrations of urolithin A (UA) for 12 h (a), 24 h (b), 48 h (c), 72 h (d), and 96 h (e) is compared with the viability of untreated cells (*P < 0.05). The cell viability was determined by a cell counting kit-8 assay.

RAW264.7 cells were treated with different concentrations of UA to determine the optimal concentration of UA, and the cytotoxic effects were determined using a CCK-8 assay. After 12 h in culture, no significant differences in the cell viability on different concentrations of UA were found (*P* > 0.05, Figure 1(a)). After 24 h or 48 h in culture, the viability of cells treated with 0, 0.5, 5, and 25 μM UA showed no significant difference compared with untreated cells (*P* > 0.05). The viability dropped only on 50 and 100 μM UA (*P* < 0.05, Figure 1(b,c)). After 72 h or 96 h, the viability of cells treated with 0, 0.5, and 5 μM UA was unaffected (*P* > 0.05) but lower than the viability of untreated cells after 25, 50, and 100 μM UA (*P* < 0.05, Figure 1(d,e)). These results suggest that 25 μM UA and 48 h are optimal treatment conditions for RAW264.7 cells in subsequent experiments.

### Effects of UA on osteoclast formation from RAW264.7 cells

**Figure 2.**
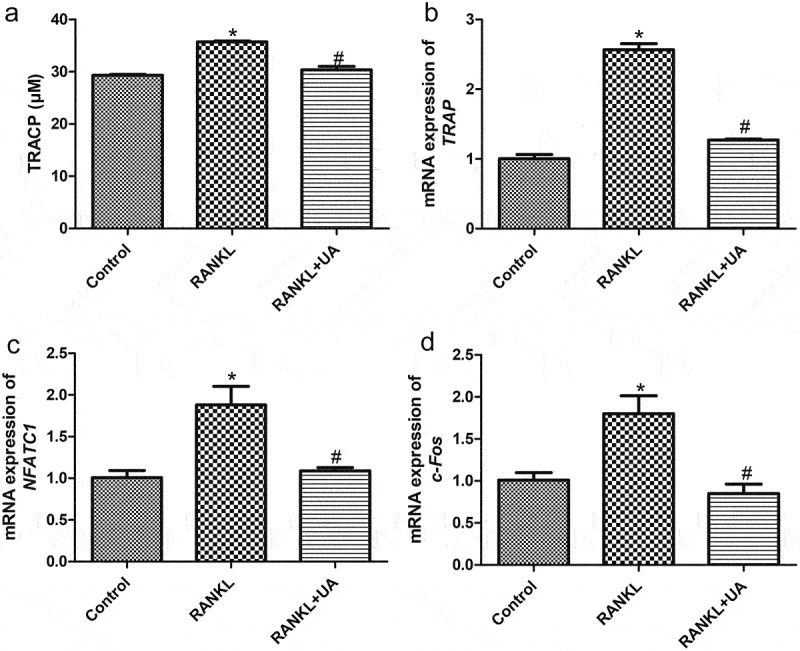
Urolithin A effects on osteoclasts differentiated from RANKL-treated RAW264.7 cells. (a) Tartrate-resistant acid phosphatase (TRACP) levels in RAW264.7 cells subject to RANKL or UA treatment. mRNA expression levels of (b) *TRAP*, **C**) *NFATC1*, and **D**) *c-Fos* in RAW264.7 cells subject to RANKL or UA treatment. *P < 0.05 represents significance compared with the control group, and ^#^*P* < 0.05, compared with the RANKL-treated group.

TRACP was quantified to investigate UA effects on osteoclast formation from RANKL-treated RAW264.7 cells. The RANKL-treated cells had significantly higher TRACP levels than the control cells (Figure 2, *P* < 0.05). When RANKL-induced cells were UA-treated, their TRACP levels dropped to those similar in the control cells (*P* > 0.05, Figure 2(a)). Next, osteoclast-specific markers *NFATC1, c-Fos*, and *TRAP* were determined by RT-qPCR. The mRNA expression of all three markers was significantly increased in RANKL-treated cells versus the control cells (*P* < 0.05). By contrast, it was markedly decreased in UA-treated RANKL-induced cells (*P* < 0.05) and similar to that of control cells (*P* > 0.05, Figure 2(b-d)). These results indicate that 100 ng/mL RANKL successfully induced the osteoclast formation from RAW264.7 cells, and 25 μM UA inhibited the RANKL-induced osteoclast formation.

### Identification and functional annotation analyses of DEGs

**Figure 3.**
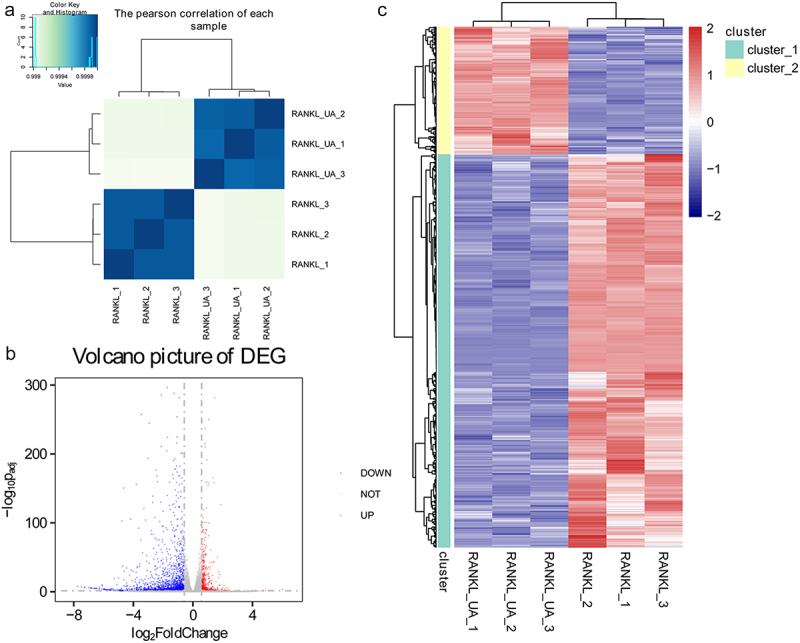
Identifying differentially expressed genes in RAW264.7 cells treated with RANKL or RANKL and UA. (a) Pearson’s correlation coefficients for each total RNA sample isolated from RANKL- or RANKL + UA-treated cell group. (b) A volcano plot of the differentially expressed genes (DEGs). The blue points represent downregulated genes, the gray points normal expression levels, and the red points upregulated genes. (c) Clustering heatmap based on the expression level of the identified DEGs.

Total RNA samples isolated from RAW264.7 cells treated with RANKL or RANKL + UA were sequenced to explore the underlying molecular mechanisms of UA on osteoclast differentiation. Based on the Pearson’s correlation analysis of each sample, we found no differences among the samples isolated from either the RANKL-treated cell group or the RANKL + UA-treated group. Conversely, we identified a significant difference between the samples from cells in the two different treatment groups (Figure 3(a)). According to the thresholds (FC > 1.5; FDR < 0.05), 2,399 DEGs were identified, of which 587 were upregulated and 1,812 downregulated (Figure 3(b)). Hierarchical clustering analysis of the identified DEGs distinguished cells in the RANKL-treated group from those in the RANKL + UA-treated group, confirming the reliability of the above results (Figure 3(c)).

**Figure 4.**
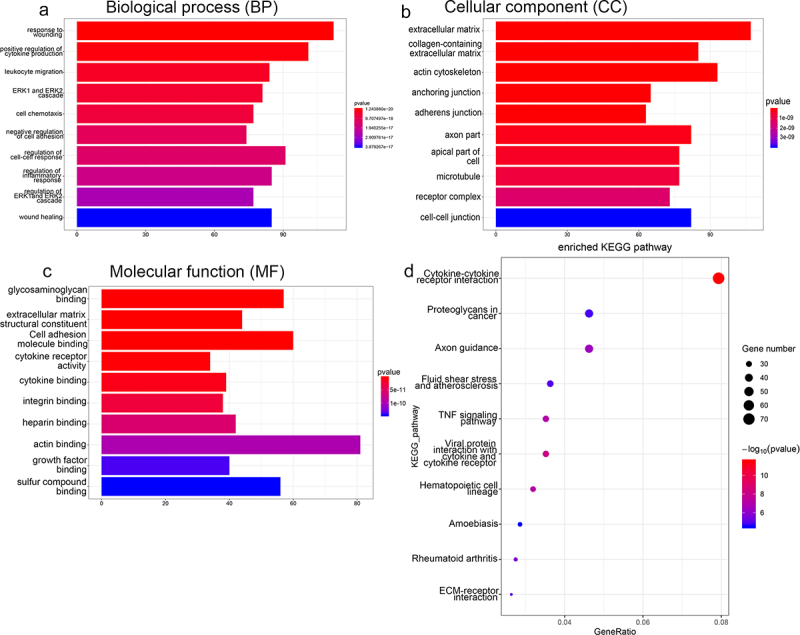
Functional annotation analyses of DEGs identified by sequencing RAW264.7 cells treated with RANKL or RANKL and UA. Gene ontology (GO) terms related to (a) biological process, (b) cellular components, and (c) molecular function are shown. (d) DEGs subject to the Kyoto Encyclopedia of Genes and Genomes (KEGG) pathway analysis are most enriched in signaling pathways related to stress, inflammation, differentiation, and disease.

The DEGs were subjected to functional annotation analyses to assess their biological identity. Figure 4 shows the most enriched GO terms belonging to biological process (BP), cellular component (CC), and molecular function (MF) terms. The DEGs associated with the BP term were implicated in response to wounding, regulation of the ERK1/2 cascade, wound healing, and regulation of the inflammatory response. Those associated with the CC term were involved in extracellular matrix, adherens junction, and microtubule functions. The DEGs associated with the MF term were involved in sulfur compound binding, glycosaminoglycan binding, actin binding, and cell adhesion molecule binding (Figure 4(a-c)). In addition, KEGG analysis found that the DEGs were significantly enriched in rheumatoid arthritis, TNF signaling pathway, fluid shear stress, atherosclerosis, and ECM-receptor interactions (Figure 4(d)).

### Verifying translatome data by RT-qPCR

**Figure 5.**
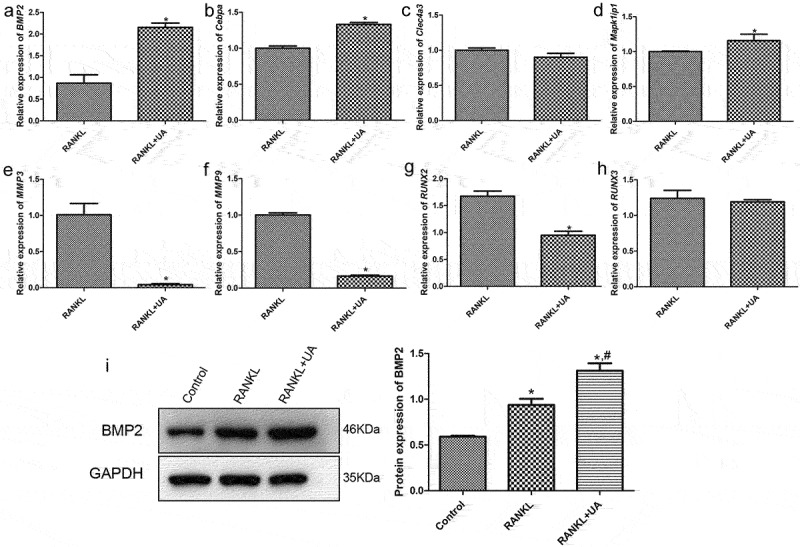
Verifying DEGs by reverse transcription-quantitative PCR. The relative mRNA expression of (a) *BMP2*, (b) *Cebpa*, (c) *Clec4a3*, (d) *Mapk1ip1*, (e) *MMP3*, (f) *MMP9*, (g) *RUNX2*, and (h) *RUNX3* in RANKL- or RANKL + UA-treated RAW264.7 cells. *P < 0.05 depicts significance compared with the RANKL-treated cell group. (i) The BMP2 protein expression detected by Western blotting. *P < 0.05 is significance compared with the control group; ^#^*P* < 0.05, with the RANKL-treated group.

Four activated (*BMP2, Cebpa, Clec4a3*, and *Mapk1ip1*) and four repressed (*MMP3, MMP9, RUNX2*, and *RUNX3*) DEGs were quantified by RT-qPCR to validate the sequencing data. The expression levels of *BMP2, Cebpa*, and *Mapk1ip1* were significantly increased in RANKL + UA-treated cells compared with those in RANKL-treated cells (*P* < 0.05, Figure 5(a,b,d)). By contrast, the expression levels of *MMP3, MMP9*, and *RUNX2* were markedly decreased in these cells compared with those treated only with RANKL (*P* < 0.05, Figure 5(e-g)). These results are thus consistent with the expression pattern observed in the sequencing data. However, no significant differences were observed in *Clec4a3* and *RUNX3* expression between treated cells (*P* > 0.05, Figure 5(c,h)). Collectively, these findings imply that the consistency rate between the RT-qPCR and sequencing analysis is 75%, which indicates a relatively high reliability of the sequencing data [23,24]. Furthermore, Western blotting showed that BMP2 was significantly upregulated in the RANKL-induced cells (*P* < 0.05) versus the control cells. Moreover, UA further increased BMP2 expression in the RANKL-treated cells (Figure 5(i)). Therefore, the role of BMP2 in osteoclast differentiation was further investigated.

### Cell transfection efficiency and scanning electron microscopy analysis

**Figure 6.**
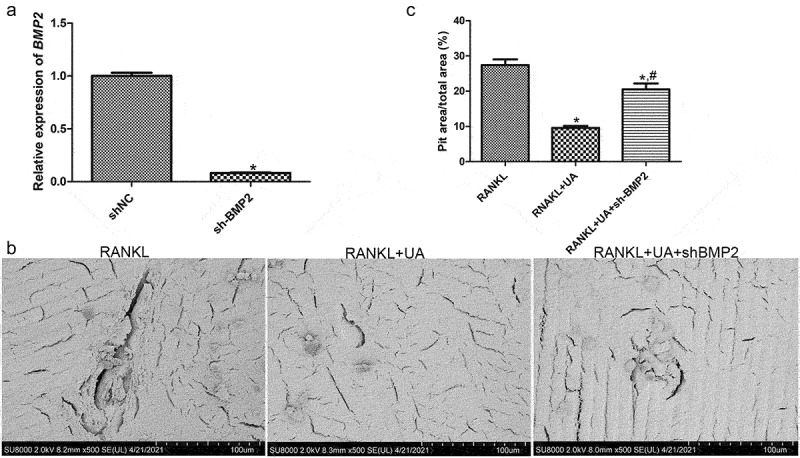
Effects of BMP2 on bone metabolism determined by gene silencing and scanning electron microscopy. (a) Cell transfection efficiency of sh*BMP2*-RAW264.7 cells was assessed by quantifying *BMP2* expression with RT-qPCR. *P < 0.05 shows significance compared with shNC-RAW264.7 cells (negative control). (b) Representative scanning electron images of bone biopsy specimens on RANKL and UA treatments in the presence of sh*BMP2*-RAW264.7 or shNC-RAW264.7 cells. (c) The bone biopsy area was quantified using the ImageJ software. *P < 0.05 represents significance compared with the RANKL-treated group; ^#^*P* < 0.05, compared with the RANKL + UA-treated group.

A *BMP2*-specific shRNA was used to silence *BMP2* in RAW264.7 cells to delineate BMP2 effects on osteoclast formation. The relative expression of *BMP2* was considerably lower in the shNC-RAW264.7 cells (negative control) than in the sh*BMP2*-RAW264.7 cells (*P* < 0.05, Figure 6(a)), suggesting that stably transfected sh*BMP2*-RAW264.7 cells were successfully established.

Bone biopsy specimens in the presence of shNC-RAW264.7 or sh*BMP2*-RAW264.7 cells were treated with RANKL or RANKL + UA and imaged with scanning electron microscopy to investigate the effects of *BMP2* on bone metabolism. In the RANKL-treated shNC cell group, the biopsy specimens showed many shallow, rough, and scattered bone resorption pits with varied sizes and clear boundaries (Figure 6(b)). In the RANKL + UA-treated shNC group, by contrast, few bone resorption pits in the specimens were visible. Remarkably, in the RANKL + UA-treated sh*BMP2* group, bone resorption pits were again more abundant. Quantitative analysis confirmed that the bone resorption area significantly decreased after UA treatment compared with that after RANKL treatment only (*P* < 0.05), clearly increasing in the RANKL + UA-treated sh*BMP2* group, relative to that in the RANKL + UA-treated shNC group (*P* < 0.05, Figure 6(c)). These results indicate that UA attenuates osteoclast-driven bone resorption, whereas *BMP2* silencing counteracts this effect.

### Western blotting analysis

**Figure 7.**
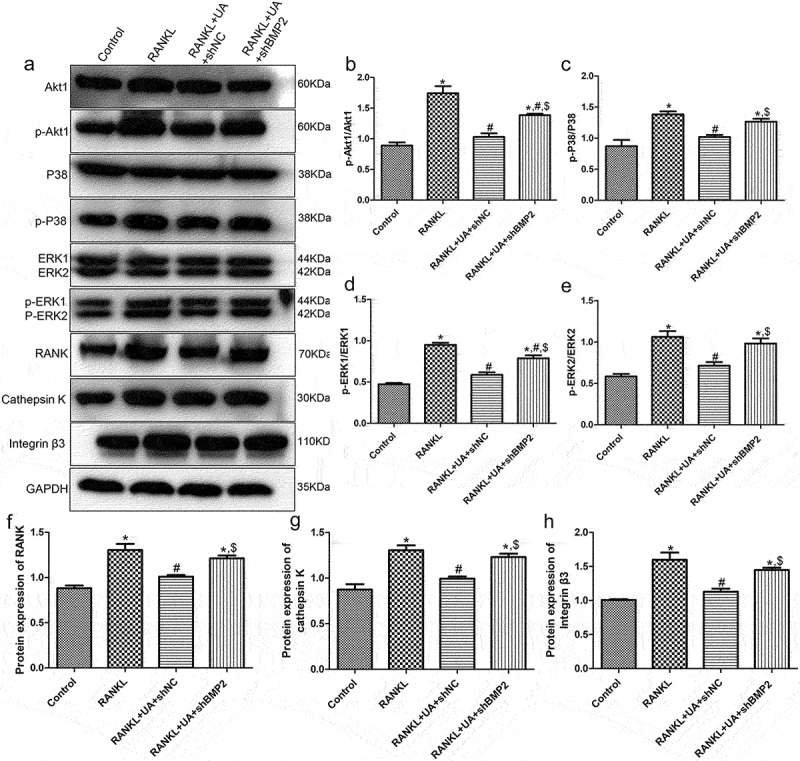
Protein expression levels of Akt1/p38/ERK pathway-related proteins and osteoclastic proteins RANK, cathepsin K, and integrin β3 detected by Western blotting. (a) Representative Western blot images. The levels of (b) phosphorylated (p)-Akt1/Akt1, (c) p-P38/P38, (d) p-ERK1/ERK1, (e) p-ERK2/ERK2, (f) RANK, (g) cathepsin K, and (h) integrin β3 are shown. *P < 0.05 shows significance compared with the untreated shNC group; ^#^*P* < 0.05, with the RANKL-treated shNC group; ^$^*P* < 0.05, with the RANKL + UA-treated shNC group.

Protein expression levels of Akt1/P38/ERK pathway-related proteins and osteoclastic proteins (RANK, cathepsin K, and integrin β3) were assessed by Western blotting in RANKL or RANKL + UA-treated cells. Representative Western blot images are summarized in Figure 7(a). No significant differences in the levels of p-Akt1/Akt1, p-P38/P38, p-ERK1/ERK1, p-ERK2/ERK2, RANK, cathepsin K, or integrin β3 between the control and RANKL + UA-treated shNC cell group were observed (*P* > 0.05, Figure 7). The levels of p-Akt1/Akt1 and p-P38/P38 were significantly higher in the RANKL- and RANKL + UA-treated sh*BMP2* group than in the control group (*P* < 0.05, Figure 7(b,c)). UA decreased the levels of RANKL-activated p-Akt1/Akt1 and p-p38/p38 (*P* < 0.05), but after *BMP2* knockdown, their levels rose again compared with those in the RANKL + UA-treated shNC group (*P* < 0.05, Figure 7(b,c)). In addition, the levels of p-ERK1/ERK1 and p-ERK2/ERK2 were similar to Akt1/p38 pathway-related protein levels across the groups (Figure 7(d,e)). The expression levels of RANK, cathepsin K, and integrin β3 were significantly higher in the RANKL-treated group than in the control group (*P* < 0.05), and UA significantly decreased their RANKL-induced expression (*P* < 0.05, Figure 7(f–h)). When *BMP2* was silenced, it considerably increased the protein levels in the RANKL + UA-treated shNC group (*P* < 0.05, Figure (f-h)).

## Discussion

Osteoporosis is a systemic bone disease that increases the susceptibility to fracture and may be caused by excessive bone resorption due to increased osteoclast activity [25,26]. UA is a gut-microbial metabolite derived from pomegranate ellagitannins and has a protective effect against osteoarthritis development in vivo and in vitro [27,28]. Pomegranate peel extract prevents bone microarchitecture impairment and loss of bone mineral density in osteoarthritis. It also stimulates osteoblastic differentiation in vitro, alleviating osteoporosis[29]. Chen et al.[30] demonstrated that UA prevents brain aging caused by D-gal through the miR-34a-mediated activation of the SIRT1/mTOR signaling pathway, indicating its neuroprotective role. Therefore, we speculated that UA regulates osteoclast activity and the progression of osteoporosis. TRACP is a marker for bone resorption and osteoclast activity[31], which are important for understanding bone metabolism under physiological conditions and various pathological conditions[32]. Sekiguchi et al.[33] reported that mangiferin significantly reduces the formation of TRACP-positive multinuclear cells, which indicates that mangiferin inhibits osteoclast differentiation. *TRAP, NFATC1* and *c-Fos* are osteoclast-specific gene markers[34]. Our study showed that RANKL significantly increased TRACP and transcript levels of all three markers relative to those in the control cells. However, UA treatment reverted their RANKL-induced levels almost to those of control cells. Taken together, RANKL promotes differentiation of RAW264.7 cells into osteoclasts, whereas UA suppresses it.

Total RNA was isolated from cells subject to RANKL treatment or the combined treatment with UA and sequenced to understand how UA inhibits RANKL-driven osteoclast differentiation. The transcriptome dataset revealed 2,399 DEGs, of which 587 were induced and 1,812 repressed. Among the notable activated genes were *BMP2, Cebpa*, and *Mapk1ip1. BMP2* is a potent growth factor that regulates osteoblast and osteoclast formation. Durbano et al.[35] indicated more significant differences in BMP signaling pathways in patients diagnosed with osteoporosis than in healthy control individuals. *Cebpa* is a key regulator of osteoclast lineage stereotyping, and its deficiency in osteoclasts can prevent bone loss[36]. Furthermore, *Mapk1ip1* expression is increased in osteosarcoma cells[37] but never assessed in osteoporosis. The repressed DEGs included *MMP3, MMP9*, and *RUNX2. MMP3* and *MMP9* are upregulated in chondrocytes in osteoarthritis[38]. Transcription factor *RUNX2* is critical for osteoblast differentiation and chondrocyte maturation and usually upregulated in chondrocytes in osteoarthritis[39]. Collectively, these differentially expressed genes, especially *BMP2*, may be involved in suppressing RANKL-induced osteoclast differentiation by UA.

Functional annotation analyses showed that the identified DEGs were associated with rheumatoid arthritis, the regulation of the ERK1/2 cascade, ECM-receptor interactions, the regulation of inflammatory response, and the TNF signaling pathway. The ERK1/2 cascade, for example, regulates various processes, such as cell proliferation, differentiation, adhesion, migration, and cell cycle progression[40]. Activated T and B cells produce pro-osteoclastogenic factors, such as TNF-α, NF-κB, RANKL, and IL-17A, accelerating bone loss in inflammatory states [15,41]. In the absence of estrogen, activated T cells secrete IL-17A, RANKL, and TNF, enhancing bone resorption and causing postmenopausal osteoporosis[15]. Rheumatoid arthritis is an inflammatory disease that predisposes patients to osteoporosis[42]. Zhou et al. identified 3,179 DEGs in elderly patients with osteoporosis, non-osteoporotic elderly controls, and non-osteoporotic young donors. They found that these genes are enriched in ECM-receptor interactions, indicating they may be related to osteoporosis[43]. Furthermore, TNF-α increases the expression of RANKL and sclerostin in osteocytes and promotes osteoclast formation[44]. These findings and our sequencing results imply that UA may relieve osteoclast differentiation by regulating multiple signaling pathways. However, their specific regulatory roles in the UA treatment of osteoporosis require further investigation.

BMP2 induces osteogenic differentiation in various cells. In mesenchymal stem cells, it causes chondrogenic differentiation and endochondral ossification. It is also essential for bone engineering[45]. Long non-coding RNA *MSC-AS1* promotes osteogenic differentiation by binding *miR-140-5p* to upregulate BMP2, relieving osteoporosis[46]. In addition, high BMP2 expression enhances osteoclast-mediated bone resorption through the classical BMP signaling pathway and p65 signaling pathway[47]. These findings suggest the importance of BMP2 in osteoclasts and osteoporosis. In our study, BMP2 expression was considerably higher in the RANKL + UA-treated RAW264.7 cells than in the RANKL-treated. Thus, BMP2 was selected as a candidate for further investigation. RANKL-treated RAW264.7 cells formed bone resorption pits in bone biopsy specimens, but UA inhibited their formation. Remarkably, BMP2 knockdown restored bone resorption. Taken together, UA suppresses osteoclast differentiation and bone resorption by regulating BMP2 expression.

Akt1 is an overactive proto-oncogene in most cancers[48]. It is also a crucial promoter of osteoblast–osteoclast coupling[49]. Activated p38 phosphorylates osteoblast- or osteoclast-related transcription factors and triggers other pathways by activating cytoplasmic substrates, such as Akt and pMK2[50]. ERK1 and ERK2, both expressed in osteoblasts, have essential roles in the Ras-RAF-MEK-ERK signal transduction cascade[51]. This cascade regulates multiple processes, including cell cycle progression, differentiation, osteoclastogenesis, metabolism, and transcription[52]. Fowler et al.[53] demonstrated that activin A completely inhibits RANKL-stimulated osteoclast differentiation, migration, and bone resorption via SMAD2, Akt1, and IκB signaling. Another study reported that Rev-erbα negatively mediates osteoblast and osteoclast differentiation by suppressing the p38 MAPK pathway[54]. Furthermore, Yue et al.[55] showed that prostaglandin D_2_ promotes osteoclast apoptosis through the ERK1/2 and Akt signaling pathways. We conclude that UA represses the RANKL-induced osteoclast differentiation of RAW264.7 cells by regulating Akt1, p38, and ERK1/2 signaling pathways, and that BMP2 may reverse the UA inhibitory effect via these pathways.

RANKL/RANK signaling activates a central regulator of osteoclast generation NFATc1, inducing osteoclast gene expression[56]. Cathepsin K is highly expressed in osteoclasts, and its inhibitors have been developed for osteoporosis management[57]. Integrin β3 regulates cell adhesion and contributes to differentiation, promoting bone formation[58]. Zeng et al.[59] found that aconine decreases the levels of the osteoclast-specific genes integrin β3 and cathepsin K, repressing RANKL-induced osteoclast differentiation. Therefore, UA treatment may inhibit RANKL-induced osteoclast formation by downregulating RANK, cathepsin K, and integrin β3, whereas BMP2 may reverse this effect.

## Conclusions

We identified 2,399 differentially expressed genes in UA-promoted inhibition of osteoclast differentiation. UA may suppress the RANKL-induced osteoclast differentiation by regulating Akt1, p38, and ERK1/2 signaling pathways and repressing osteoclast-related proteins. Additionally, BMP2 was shown to be a novel UA target in inhibiting osteoclast differentiation. However, the regulatory roles of the identified genes, except *BMP2*, and the signaling pathways require further investigation. The potential therapeutic effects of UA on osteolytic diseases require further confirmation by culturing osteoblasts with conditioned medium from osteoclast cultures and in vivo experiments. Our results uncover the role of UA in regulating osteoclast activity and provide insights into the underlying mechanisms. Moreover, our findings propose BMP2 as a potential drug target for the therapy of bone metabolic diseases, such as osteoporosis, based on its roles in osteoclast differentiation.

## Data Availability

The dataset used and/or analyzed during the current study are available from the corresponding author on reasonable request.

## Highlights

- UA inhibition of osteoclast differentiation involves 2,399 DEGs.
- UA suppresses RANKL-induced osteoclast differentiation via Akt1, p38, and ERK1/2.
- BMP2 is a novel UA target for inhibiting osteoclast differentiation.
